# BDNF and KISS-1 Levels in Maternal Serum, Umbilical Cord, and Placenta: The Potential Role of Maternal Levels as Effect Biomarker

**DOI:** 10.1007/s12403-023-00565-w

**Published:** 2023-05-29

**Authors:** Sebastian Granitzer, Raimund Widhalm, Simon Atteneder, Mariana F. Fernandez, Vicente Mustieles, Harald Zeisler, Markus Hengstschläger, Claudia Gundacker

**Affiliations:** 1 Institute of Medical Genetics, Medical University of Vienna, Waehringer Strasse 10, 1090 Vienna, Austria; 2 Center for Biomedical Research (CIBM), University of Granada, Granada, Spain; 3 Instituto de Investigación Biosanitaria (Ibs.GRANADA), Granada, Spain; 4 Consortium for Research and Public Health (CIBERESP), Instituto de Salud Carlos III, Madrid, Spain; 5 Department of Obstetrics and Gynecology, Medical University of Vienna, Vienna, Austria; 6 Exposome Austria, Research Infrastructure and National EIRENE Hub, Vienna, Austria

**Keywords:** Pro-BDNF, Mature BDNF, Kisspeptin, Biomarker, Neurodevelopment, Placenta

## Abstract

Brain-derived neurotrophic factor (BDNF) and kisspeptin-1 (KISS-1) regulate placental development and fetal growth. The predictive value of maternal serum BDNF and KISS-1 concentrations for placental and umbilical cord levels has not yet been explored. The influence of prenatal lead (Pb) and cadmium (Cd) exposure and maternal iron status on BDNF and KISS-1 levels is also unclarified and of concern. In a pilot cross-sectional study with 65 mother–newborn pairs, we analyzed maternal and cord serum levels of pro-BDNF, mature BDNF, and KISS-1, BDNF, and KISS-1 gene expression in placenta, Pb and Cd in maternal and umbilical cord blood (erythrocytes), and placenta. We conducted a series of in vitro experiments using human primary trophoblast cells (hTCs) and BeWo cells to verify main findings of the epidemiological analysis. Strong and consistent correlations were observed between maternal serum levels of pro-BDNF, mature BDNF, and KISS-1 and corresponding levels in umbilical serum and placental tissue. Maternal red blood cell Pb levels were inversely correlated with serum and placental KISS-1 levels. Lower expression and release of KISS-1 was also observed in Pb-exposed BeWo cells. In vitro Pb exposure also reduced cellular BDNF levels. Cd-treated BeWo cells showed increased pro-BDNF levels. Low maternal iron status was positively associated with low BDNF levels. Iron-deficient hTCs and BeWo cells showed a consistent decrease in the release of mature BDNF. The correlations between maternal BDNF and KISS-1 levels, placental gene expression, and umbilical cord serum levels, respectively, indicate the strong potential of maternal serum as predictive matrix for BDNF and KISS-1 levels in placentas and fetal sera. Pb exposure and iron status modulate BDNF and KISS-1 levels, but a clear direction of modulations was not evident. The associations need to be confirmed in a larger sample and validated in terms of placental and neurodevelopmental function.

**Supplementary Information:**

The online version contains supplementary material available at 10.1007/s12403-023-00565-w.

## Introduction

Within the European Human Biomonitoring Initiative (HBM4EU—https://www.hbm4eu.eu/), a series of comprehensive reviews were performed to identify promising effect biomarkers (i.e., markers of health-disease), in order to complement human exposure data with mechanistically based biomarkers of early adverse effects in human biomonitoring (HBM) studies. Guided by toxicological and adverse outcome pathway (AOP) information, promising effect biomarkers were prioritized, including brain-derived neurotrophic factor (BDNF) and kisspeptin-1 (KISS-1), related to neurodevelopmental and reproductive outcomes, respectively (Gundacker et al. 2021a; Mustieles et al. 2020). Recent data in HBM4EU-funded studies have shown that exposure to heavy metals is associated with altered serum BDNF regulation and behavior among adolescent boys from the INMA-Granada cohort Rodriguez-Carrillo et al. (2022b) and that blood BDNF regulation even mediated the association between exposure the endocrine disruptor bisphenol A (BPA) and behavioral problems in this same cohort (Mustieles et al. 2022). Additionally, preliminary data are also revealing an influence of environmental chemicals including heavy metals on serum KISS-1 regulation among adolescent boys (Rodriguez-Carrillo et al. 2022c). Beyond these promising results, many gaps still remain around the physio- and pathophysiology of BDNF and KISS-1 regulation, especially during critical developmental periods such as pregnancy.

BDNF is a member of the neurotrophin family of growth factors, which is important for fetal brain development, especially synaptic plasticity (Binder and Scharfman 2004; Cohen-Cory et al. 2010). BDNF is highly conserved in gene structure and function (Gotz et al. 1992). Its spatio-temporal expression is tightly regulated through transcriptional, post-transcriptional, and epigenetic mechanisms (Hing et al. 2018). In addition to (epi)genetic influences, environmental pollutants such as lead (Pb) can affect BDNF levels (Gundacker et al. 2021a).

BDNF expression can be constitutive (e.g., in neurons) or on demand, depending on the cell type. It is synthesized as a precursor form, pro-BDNF that is converted to mature BDNF via intracellular factors (furin or intracellular convertases) or extracellular factors (plasmin and metalloproteinases MMP2 and MMP9) by proteolytic cleavage. Both isoforms fulfill fundamentally opposite functions, by interacting with different receptors. While pro-BDNF induces apoptosis by binding to the p75 neurotrophin receptor, mature BDNF promotes cell survival and synaptic plasticity by activating the tyrosine kinase receptor (Kowianski et al. 2018). Changes in brain morphology, motoric impairments, and even reduced lifespan in a pro-BDNF knockout model with drastically reduced levels of both isoforms, clearly demonstrate the significant role of BDNF during development (Li et al. 2020).

Although BDNF levels are highest in brain, it is expressed in over 20 different human tissues, including placenta (Pruunsild et al. 2007). Furthermore, BDNF can also be found circulating in blood, examined as a potential biomarker for psychiatric disorders, as reduced serum BDNF levels have been associated with various mental disorders (e.g., depression, schizophrenia) (Cattaneo et al. 2016; Rodrigues-Amorim et al. 2018). The major functions of BDNF include developmental processes, regulation of neurogenesis, gliogenesis, synaptogenesis, and neuroprotection (Gonzalez et al. 2016; Sasi et al. 2017). In addition, BDNF has specific roles during pregnancy, as it is essential for follicular development, implantation, and placentation (Kawamura et al. 2007, 2009; Mayeur et al. 2010).

KISS-1, also known as metastin (due to its identification as metastasis suppressor in melanoma), is a peptide hormone, which collectively describes different circulating isoforms (kisspeptin-54, -13, -14, and -10) generated through different proteolytic cleavage sites. By binding to G-protein-coupled receptor (GPR54/KISS-1R), kisspeptins regulate in addition to tumor metastasis, puberty onset, and fertility through stimulation of gonadotropin and gonadotropin-releasing hormone (GnRH), as well as trophoblast invasion during pregnancy (Zhu et al. 2022). Next to key limbic brain regions (e.g., hypothalamus), KISS-1 is also highly expressed in pancreas and placenta (Mills et al. 2022). While circulating KISS-1 levels are low in males and non-pregnant women, they gradually increase during pregnancy (up to 9000-fold in third trimester compared to non-pregnant individuals) and rapidly decline post-delivery (Horikoshi et al. 2003).

KISS-1 is known to play an important role in human development in the initiation and progression of puberty (Tng 2015). Due to its high expression and important role in placenta, it has been suggested that KISS-1 could be a biomarker of pregnancy complications (Tsoutsouki et al. 2022). However, the physiological role of circulating KISS-1 in pregnancy remains unknown (Reynolds et al. 2009). Of note, both BDNF and KISS-1 are expressed in the human placenta regulating its development and fetal growth with implications for offspring health (Dingsdale et al. 2021; Hu et al. 2019; Kapustin et al. 2020; Mayeur et al. 2010; Radovick and Babwah 2019; Rosenfeld 2020). The concept of the placenta–brain axis describes the close connection between the placenta and brain development (Rosenfeld 2020). The placenta not only supplies the fetus with nutrients and gases, but also releases neurotransmitters (serotonin, dopamine, noradrenaline/epinephrine) that can influence brain development. For example, neurobehavioral disorders such as autism spectrum disorders, have been attributed to placental dysfunctions (Rosenfeld 2020).

Male fetuses have been shown to have lower BDNF serum levels in the umbilical cord than female fetuses (Dingsdale et al. 2022). Low neonatal serum BDNF levels have been associated with impaired neurodevelopment (Dingsdale et al. 2022; Skogstrand et al. 2019; Yu et al. 2016), i.e., in cases with neurodevelopmental disorders (e.g., autism), lower BDNF serum levels have been found in umbilical cord serum than in healthy controls (Skogstrand et al. 2019; Yu et al. 2016).

Therefore, a marker that early and reliably indicates changes in placental BDNF/KISS-1 expression and/or fetal serum levels would be of great value for both environmental epidemiology and clinical fields. However, the predictive value of maternal serum BDNF and KISS-1 concentrations and their relationship to placental and umbilical cord serum BDNF levels has not yet been explored. The possible influences of prenatal Pb and cadmium (Cd) exposure and maternal iron status on BDNF and KISS-1 levels have neither been elucidated.

The objectives of the study were to (1) determine whether maternal serum BDNF and KISS-1 levels are correlated with umbilical cord serum levels and placental gene expression; (2) determine whether BDNF and KISS-1 levels can be used as biomarkers of effect in relation to Pb and Cd exposure; and (3) evaluate the influence of maternal iron status on BDNF and KISS-1 levels. Most experiments were conducted in choriocarcinoma cell line BeWo and the main findings confirmed in human primary trophoblast cells isolated from placentas.

## Materials and Methods

### Study Design and Sample Collection

Sixty-five pregnant women were recruited at the General Hospital Vienna between July 2019 and October 2021 in the frame of the placental iron metabolism study (Ethic Vote No. 1404/2015) and included in this cross-sectional pilot study after providing written informed consent. Further inclusion criteria were healthy single term pregnancy, delivered between GW 37 and 42 and age between 18 and 45 years. Some pregnant women that conceived by in vitro-fertilization, having allergies or taking iron supplements were included. Women with COVID-19 infection during pregnancy, bariatric surgery, hypo- and hyperthyroidism, and medications that may affect iron metabolism (e. g., heparins) were excluded.

Participants were interviewed by trained staff around birth. Maternal venous blood samples were collected prior to child delivery. After birth, venous umbilical cord blood was drawn and placentas collected. Full thickness pieces were taken from four sites of the abembryonic pole of the placenta (1 sample per quadrant), all four were homogenized together to represent the whole placenta and stored at − 20 °C until further analyses. An aliquot of the four approximately 3 mm^3^ pieces of the placenta was stored in RNAlater® Solution (ThermoFisher) at − 20 °C for gene expression analyses by qPCR.

Most blood samples were analyzed for blood counts and iron status (Table 1). Maternal iron supplementation was surveyed in three categories, i.e., no supplementation, supplementation with multivitamin/multielement supplements (containing iron), and intentional supply of iron, mostly via oral iron preparations. Nearly 90% of women received some form of iron supplementation, i.e., 45% received iron-containing dietary supplements and 44% received intentional iron therapy by ferrous sulfate supplements (100–160 mg/day).

**Table 1:** Study group characteristics

	*N* (%)	Median	AM	SD	Min	Max
Pregnant women						
Maternal age (years)	65	36.0	34.6	5.5	22.0	45.0
Gestational length (days)	65	271	270	7.2	249	288
Height (cm)	65	165	166	5.9	150	180
Pre-pregnancy BMI	65	23.1	24.6	5.3	17.0	39.8
Pregnancy BMI	65	27.6	28.2	5.8	21.3	44.1
Hemoglobin (g/dl)	57	11.6	11.7	1.0	9.5	14.6
Hematocrit (%)	57	34.0	34.5	2.9	28.6	43.4
Ferritin (µg/l)	57	24.3	34.8	33.7	14.9	216.4
Transferrin saturation (%)	57	17.8	19.4	11.3	5.0	73.3
Transferrin (mg/dl)	57	353	364	67.4	148	545
sTFR (mg/dl)	57	1.24	1.35	0.50	0.60	2.90
Serum iron (µg/dl)	56	90.5	90.7	36.6	31.0	191
Ery-Pb (µg/kg)	60	29.9	33.7	18.8	5.1	100.5
Newborn						
Birth weight (g)	65	3297	397	442	2480	4830
Female	30 (46)					
Males	35 (54)					
Hemoglobin (g/dl)	56	15.2	15.2	1.5	10.9	19.2
Hematocrit (%)	56	44.9	44.7	4.5	31.7	55.7
Ferritin (µg/l)	56	199	209	113	15.0	521
Transferrin saturation (%)	56	73.9	67.9	17.4	21.7	92.6
Transferrin (mg/dl)	56	154	163	34.9	96.0	261
sTFR (mg/dl)	56	1.67	1.80	0.50	1.10	3.50
Serum iron (µg/gl)	56	150	151	36.8	80.0	220
Ery-Pb (µg/kg)	62	24.0	32.4	28.7	4.14	152
Plac-Cd (µg/kg)	65	3.61	4.42	2.78	1.52	15.9

Pb was not detected in any placenta samples. Cd was detected in only eight samples of maternal erythrocytes and in none of the cord erythrocytes. Missing Ery-Pb levels were < LOD

*AM* arithmetic mean, *BMI* body mass index, *sTFR* soluble transferrin receptor, *Ery-Pb* erythrocyte lead (Pb) levels, *Plac-Cd* placental cadmium (Cd) level

Cd and Pb levels were determined in erythrocytes (i.e., red blood cell fraction) and placentas (homogenate of four tissue aliquots). Medical records were used to survey maternal anthropometry, medical history, and health status during pregnancy and delivery, as well as neonatal birth outcomes (birth weight, birth length, head circumference, and gestational age). Iron status was characterized by various parameters (Table 1).

The participants also completed a Food-Frequency-Questionnaire (FFQ) that contained mainly single-choice questions focusing on iron rich foods, vitamin-C-rich foods or foods that prevent intestinal iron absorption. To calculate estimated daily iron intake (mg/day), each food was assigned an iron content (obtained from https://www.oenwt.at/), which was normalized by portion/serving size.

### ELISA

Mature BDNF and pro-BDNF were quantified in both human serum and cell culture supernatants in duplicates using the Mature BDNF/pro-BDNF Combo Rapid ELISA Kit (Biosensis) according to the manufacturer's protocol. Serum samples were diluted 1:10 for pro-BDNF, 1:50 for mature BDNF, and 1:2 for cell culture supernatants to be within the detection range of the ELISA assay (0–1000 pg/ml). The concentrations of the reference material for pro-BDNF (463 ± 14.7 pg/ml) as well as for mature BDNF (262 ± 7.5 pg/ml) were well within the certified range (pro-BDNF: 350–650; mature BDNF: 175–325 pg/ml).

KISS-1 was quantified by KISS-1 Metastasis Suppressor (KISS-1) ELISA Kit (Antibodies-online, ABIN6962789), according to the manufacturer's protocol. Human serum samples were diluted 1:10 and 1:2 for cell culture supernatants to be within the detection range of the KISS-1 ELISA assay (0–1000 pg/ml).

Intra- and inter-plate CV was also determined for pro-BDNF (intra-assay CV: 3.2%; inter-assay CV: 3.1%), as well as for mature BNDF (intra-assay CV: 3.8%; inter-assay CV: 2.9%) and for KISS-1 (intra-assay CV: 3.8%; inter-assay CV: 4.3%).

Commercially available ELISA kits may give different results depending on the company from which they are purchased (Polacchini et al. 2015). For this reason, all ELISA kits we have used are from the same company and the same LOT.

### RNA Isolation, Reverse Transcription, and qPCR

RNA from whole placental tissue stored in RNAlater was isolated using PARIS™ Kit (Invitrogen™), while cellular RNA was isolated using TRI®Reagent (Merck) according to the manufacturer’s instructions, respectively. Total RNA was reverse transcribed using Go-Script Reverse Transcription System (Promega). cDNA was diluted 1:10, and 2 µl cDNA solution was used as template in gene expression assay reactions, following Applied Biosystems StepOnePlus Real-Time PCR System protocol. The employed primers were Hs00158486_m1 (KISS-1), Hs02718934_s1 (BDNF), Hs04194247_g1 (MT2A), and as housekeeping gene Hs00824723_m1 (Ubiquitin C: UBC) (ThermoFisher).

### Lead and Cadmium Levels in Erythrocytes and Placenta

About 1 g of erythrocytes (corresponds to approx. 2.5 ml whole blood) and placenta tissue as well as reference material (Trace Elements Whole Blood L-2, 210205, Seronorm™) were digested with 1.5 ml nitric acid (69%; Suprapur; Roth) in a microwave (MARS6, CEM) and analyzed for Cd and Pb by graphite-furnace atomic absorbance spectroscopy (GF-AAS; Zeenit-P700; Analytic Jena). The mean concentrations of ten reference material samples (Cd: 4.4 ± 0.3 µg/l, 87 ± 7% recovery; Pb: 279 ± 32 µg/l, 92 ± 11% recovery) were within the certified range (Cd: 4.1–6.1 µg/l; Pb: 272–334 µg/l). The instruments’ mean limit of detection (LOD) was 0.04 ± 0.01 µg/l for Cd and 0.84 ± 0.17 µg/l for Pb (*N* = 5). The mean limit of quantification (LOQ) was 0.13 ± 0.04 µg/l for Cd and 2.51 ± 0.50 µg/l for Pb (*N* = 5). All samples were measured in duplicate [relative standard deviation (RSD) < 10%], and the concentrations were calculated from a standard curve (0–7.5 µg/l Cd; 0–50 µg/l Pb).

### Cell Culture

Human trophoblast cells (hTCs) were isolated from healthy placentas according to previous studies (Straka et al. 2016). Isolated primary hTCs were cultured in RPMI-1640 (Gibco), supplemented with 10% fetal bovine serum (FBS; PAN Biotech) and 1% keratinocyte growth supplement (Gibco, standard conditions). hTCs were cultured at 37 °C with 5% CO_2_ and 95% humidity.

BeWo cells (clone 24), donated by Isabella Ellinger’s Lab (Medical University of Vienna), were cultured in RPMI-1640 (Gibco) supplemented with 10% FBS (PAN-Biotech) and 1% Glutamax (Gibco). Cells were cultured at 37 °C with 5% CO_2_ and 95% humidity. Cell line identity and purity were verified by short tandem repeat (STR) profiling (ATCC). Cell number was determined using CASY® cell counter (Innovatis).

Cell culture media for ELISAs were centrifuged for 10 min at 800 × g to remove cell debris; supernatants were then aliquoted and stored at − 80 °C until further analysis.

### In Vitro Iron Deficiency and Iron Overload Conditions

Primary trophoblasts (1 × 10^7^/10 cm plate) or BeWo cells (4 × 10^5^/6-well) were cultivated 24 h post-seeding under standard, iron deficiency, and iron overload conditions for 72 h. Iron deficiency and overload conditions were mimicked by the use of iron chelator deferoxamine (DFO, Merck) and ferric ammonium citrate (FAC, Honeywell), respectively. Aqueous stocks were prepared, which were diluted 1:100 (DFO) or 1:1000 (FAC) in cell culture medium to achieve final working concentrations between 25 and 500 µM for DFO, and between 5 and 100 µM for FAC.

### Exposure of Placental Cells to Lead and Cadmium

BeWo cells were seeded in 6 wells at cells 4 × 10^5^/well. After 24 h cultivation, media were changed with treatment solutions containing different concentrations of Pb and Cd and cultured for 72 h. CdCl_2_ (Merck) and PbCl_2_ (Merck) were dissolved in dimethyl sulfoxide (DMSO, Merck) and diluted 1:1000 in cell culture medium to achieve final working concentrations of 0.5–5 µM CdCl_2_ and 0.5–50 µM PbCl_2_, respectively. Concentrations in in vitro experiments were based on previous studies on toxicity of Cd (Widhalm et al. 2020) and Pb (Gundacker et al. 2012) to placental cells and fibroblasts (IMR-90), respectively.

### Cell Viability and Cytotoxicity

Cell viability and cytotoxicity were determined using RealTime-Glo MT Cell Viability Assay (Promega) and CellTox Green Cytotoxicity Assay (Promega) according to the manufacturer’s protocol. Per well of white opaque 96-well plates (Corning) 1 × 10^3^ cells were seeded. Assay performance was validated with 10 µM ionomycin (Sigma) (Supplementary Fig. 1a, b).

### Apoptosis Assay

Apoptosis was determined after 72 h using Caspase-Glo 3/7 Assay System (Promega) according to the manufacturer’s protocol. Per well of white opaque 96-well plates (Corning) 1 × 10^3^ cells was seeded. Assay performance was validated with 1 µM staurosporin (Sigma, Supplementary Fig. 1c).

### GSH/GSSG Assay

Total, oxidized, and reduced GSH, as well the ratio between reduced and oxidized forms of GSH were determined after 72 h using the GSH/GSSG-Glo Assay (Promega) according to the manufacturer`s protocol. In each well of white opaque 96-well plates (Corning), 1 × 10^3^ cells were seeded.

### Statistical Analysis

Correlation analyses (Spearman correlation coefficients, *r*_S_) and group comparisons (Mann–Whitney *U* test, Kruskal–Wallis test) were used to identify variables associated with the continuous outcome variables pro-BDNF, mature BDNF, and KISS-1 levels of maternal and cord serum as well as placental total BDNF and KISS-1 expression levels. Variables that were significantly and plausibly associated with the outcome variables (*P* < 0.05, *r*_S_ > 0.10) were included into categorical regression (CATREG) analyses. CATREG assigns numerical values to categorical variables, resulting in an optimal linear regression equation for the transformed variables. It also allows ranking of predictive variables by importance using Pratt's relative importance measure. If multicollinearity was present (verified by Spearman correlation analyses), the best-fitting variable was kept. The final CATREG models were obtained upon stepwise elimination of non-significant and/or unimportant factors from the initial (crude) model by using *P* > 0.1 and/or Pratt coefficient < 0.05 as the elimination criteria. CATREG models were calculated using maternal serum BDNF and KISS-1 as outcome variables. As the models for pro-BDNF and mature BDNF provided the same results, only the results for pro-BDNF are shown.

The in vitro data represent mean values ± standard deviation (SD) from at least 3 independent experiments (three different BeWo cell passages or hTCs isolated from three to six different placentas) made in one to six technical replicates (for details see figure legends). Two-group comparisons were done by two-sided unpaired *t* test, all others by One-Way ANOVA followed by Welch’s correction.

IBM SPSS 28 and GraphPhad Prism 9 were used for statistical analyses. The critical significance level was set at *α* = 0.05.

## Results and Discussion

### Maternal Serum BDNF and KISS-1 Levels and Their Relationship to BDNF and KISS-1 Levels in Cord Serum and Placenta

Biomarkers can provide insight into biological processes, appropriate biological functionality pathogenic processes, and pharmacologic therapy responses. To do so, biomarkers must be valid (known range of conditions under which reproducible and accurate data can be assayed) and qualified (linked to the biological process or established clinical endpoint) (Wagner 2008). In the present study, we wanted to investigate whether maternal serum levels of pro-BDNF, mature BDNF, and KISS-1 could be used as biomarkers for their respective cord serum levels and with respect to placental expression of their corresponding genes, so that maternal serum levels could be useful biomarkers of placental health and even predictors of fetal (neuro)development.

BDNF is predominantly expressed in the brain, heart, and male and female reproductive tissue including placenta (Uhlen et al. 2015). Another source of BDNF release is the platelets that play a central role in the regulation of serum BDNF (Farmer et al. 2021; Fujimura et al. 2002; Le Blanc et al. 2020). However, the main source of KISS-1 in maternal serum is the placenta. KISS-1 is mainly expressed during pregnancy in the placenta followed by a much lower expression in the brain (Uhlen et al. 2015).

**Fig. 1:**
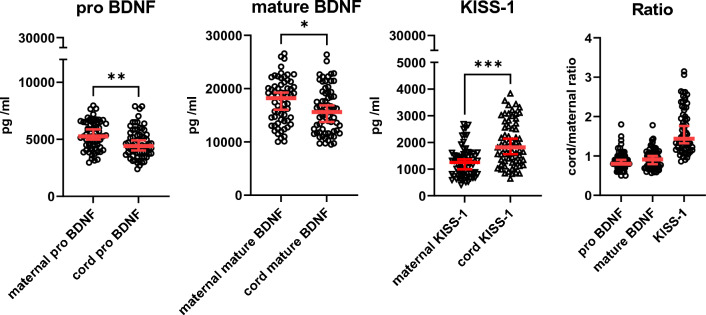
BDNF and KISS-1 concentrations of mother–newborn-pairs. Serum pro-BDNF, mature BDNF, and KISS-1 concentrations in maternal and cord serum, and the ratio of cord to maternal serum levels (Kruskal–Wallis Test *P < 0.05, **P < 0.01, ***P < 0.001; *N* = 65)

The pro and mature forms of BDNF, as well as KISS-1, could be detected in maternal and cord serum. Both, pro and mature BDNF levels, were slightly higher in maternal serum (mean pro-BDNF: 5365 pg/ml; mean mature BDNF: 17,644 pg/ml) than in cord serum (mean pro-BDNF: 4639 pg/ml; mean mature BDNF: 15,932 pg/ml). Furthermore, pro-BDNF levels were about 3 times lower than mature BDNF levels, both in maternal and cord serum (cord serum to maternal serum ratio of KISS-1: 1.65; pro-BDNF: 0.88; mature BDNF: 0.93). Lower levels were found for KISS-1, in maternal serum (mean 1265 pg/ml) than in cord serum (mean 1962 pg/ml). The ratio of cord to maternal levels was about 1, for both pro-BDNF and mature BDNF, while it approximated to 1.5 for KISS-1 (Fig. 1). No sex-specific differences were found for pro-BDNF, mature BDNF, or KISS-1 levels in cord serum (Supplementary Fig. 2).

**Fig. 2:**
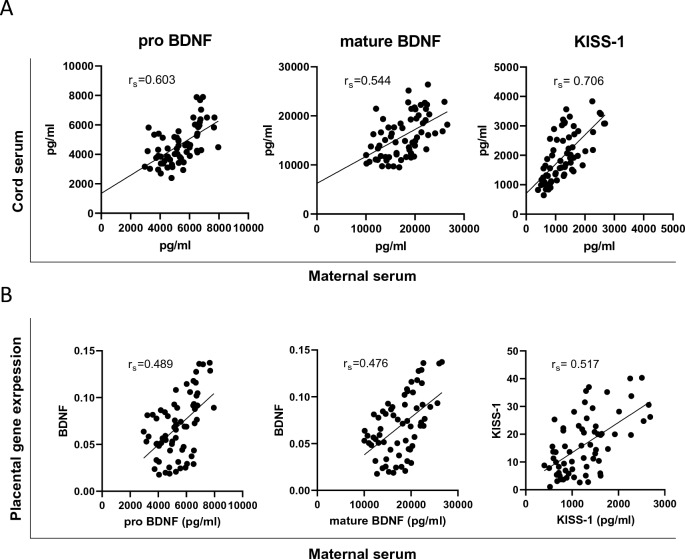
Correlations of serum levels and placental gene expression. Scatterplots of **A** maternal and cord serum levels and **B** maternal serum pro-BDNF, mature BDNF and KISS-1 and placental BDNF and KISS-1 gene expression normalized to UBC (Ubiquitin C, *N* = 65)

Maternal and cord serum levels of pro-BDNF, mature BDNF, and KISS-1 were moderately to strongly correlated (*P* < 0.05; pro-BDNF: *r*_S_ = 0.60; mature BDNF: *r*_S_ = 0.54; KISS-1: *r*_S_ = 0.71), indicating that maternal serum levels are a good proxy for cord serum levels (Fig. 2A). Similarly, moderate to strong correlations were found between pro-BDNF, mature BDNF, and KISS-1 levels in maternal serum and their respective gene expression in placental tissue (Fig. 2B; Supplementary Fig. 3).

BDNF has been proposed as a biomarker of psychiatric disorders in adults, including the occurrence of postpartum depression and other affective disorders in women with low serum BDNF levels (Dhiman et al. 2014; Gao et al. 2016; Pinheiro et al. 2012). Its potential as biomarker for impaired neurodevelopment and cognitive functions in neonates and children is unclear. However, several studies have observed associations between reduced BDNF levels in either maternal or umbilical cord serum and impaired neurodevelopment (Table 2). Our study suggests that the analysis of maternal BDNF levels could help predict placental and fetal levels, which would make it a good biomarker to address BDNF levels in these compartments.

**Table 2:** BDNF and KISS-1 levels associated with neurodevelopment

	Type of study	Population-animal model	Findings
BDNF			
Cannon et al. (2008)	Epi Study	Infants (*N* = 444)	Adults who developed schizophrenia had lower cord blood BDNF levels as infants
D’Souza et al. (2014)	Epi Study	Pregnant women (*N* = 201)	Lower maternal BDNF levels could negatively impact child neurodevelopment in preeclamptic pregnancies
Li et al. (2020)	In vivo	Pro-BDNF KO Mice	Pro-BDNF KO mice mimick a Huntington’s disease (HD)-like phenotype. Showing changed apoptosis markers in brain and performing worse in behavioral tests
Pascual-Mancho et al. (2022)	Epi Study	Pregnant women (*N* = 130)	Lower cord blood BDNF levels in FGR with Doppler alteration compared to AGA fetuses
Rao et al. (2009)	Epi Study	Infants (*N* = 33)	Cord serum BDNF levels positively correlated with postnatal neurodevelopmental scores in preterm infants
Su et al. (2021)	Epi Study	Infants (*N* = 24)	Children with lower BDNF levels and from mothers with GDM performed worse in language development (language performance correlated with serum BDNF at 12 months of age)
Yu et al. (2016)	Epi Study	Infants (*N* = 377)	Lower cord serum BDNF level correlated with poor neurodevelopment in 1-year-old children
Dingsdale et al. (2022)	Epi Study	Infants (*N* = 56)	Higher BDNF levels were associated with greater intensity of face interest
Luft et al. (2022)	In vivo	Mice	Exercise during pregnancy increased BDNF expression in the hippocampus of offspring at adult age
KISS-1			
Desroziers et al. (2012)	In vivo	Rat	Kisspeptin neurons in rat ARC arise locally during a prolonged embryonic period. There is an early functional association between kisspeptins and GnRH neurons in rat embryo
Fiorini and Jasoni (2010)	In vivo*/*In vitro	Mice	KISS-1 function is similar to neurite growth regulators
			KISS-1 induces growth of prenatal GnRH neurone
Kim et al. (2020)	In vivo	Neonatal organotypic brain slice cultures (mice)	Neonatal kisspeptin neurons of the arcuate nucleus are able to maintain a synchronous Ca^2+^ oscillation of neuronal activity
Kumar et al. (2015)	In vivo*/*In vitro	Mice	Kisspeptin neurons already communicate with GnRH neurons before birth
Zhao et al. (2014)	In vivo	Zebrafish (*Danio rerio*)	Kiss1 is the primary stimulator of GnRH3 neuronal development in ZFE and possibly coordinates maturation of zebrafish neural network

*AGA* adequate growth for gestational age, *ARC* arcuate nucleus of the hypothalamus, *FGR* fetal growth restriction, *GDM* gestational diabetes mellitus, *GnRH* gonadotropin-releasing hormone, *KO* knockout, *ZFE* zebrafish embryo

KISS-1 is known to be involved in the neurodevelopment of rodents and zebrafish (Table 2), but it is unknown whether it is also involved in this function in humans, especially in late pregnancy. Although experimental studies with KISS-1 deficient mice (whose placenta have almost no KISS-1 expression) show normal placental structures and produce healthy offspring (Herreboudt et al. 2015), it is possible that one of the functions of KISS-1 during pregnancy in humans can maintain proper placental function (Babwah 2015). Therefore, reduced levels of KISS-1 could be indicative of an impaired placenta, which would support the developing child less efficiently, increasing its risk of inadequate development. Of note, Kisspeptin has been proposed as a biomarker of miscarriage (Sullivan-Pyke et al. 2018).

### Lead and Cadmium Levels as well as Iron Status Affect BDNF and KISS-1 Levels

Pb could be quantified in maternal and cord blood erythrocytes, but not in placental tissue. In blood, Pb and Cd are mainly stored in erythrocytes and, therefore, are regarded as short-term biomarker (Malin Igra et al. 2019; Martinez-Finley et al. 2012). As there is a trend of decreasing Pb blood levels in Europe in the last decades (Gundacker et al. 2021a), this heavy metal might only marginally accumulate in placenta and its concentration thereby too low to be detected with our standard protocol but also other standard methods (e.g., Freire et al. 2019). In contrast, Cd is known to be efficiently retained in placenta (e.g., Kippler et al. 2010) and was detectable in all placenta samples, but found in only eight samples of maternal erythrocytes and in none of the cord erythrocytes (Table 1). The low to undetectable levels of Cd in blood suggest that recent exposure has been very low. Urinary Cd has been described as a measure of long-term exposure (Adams and Newcomb 2014) and, therefore, might have been a more informative exposure marker. Overall, the Pb and Cd values were within the range reported in previous studies and reviews (Esteban-Vasallo et al. 2012; Gundacker et al. 2021b; Gundacker and Hengstschläger 2012; Kelly et al. 2013). Maternal supplementation with vitamin preparations containing calcium and zinc may result in less Cd being absorbed, thus, leading to lower Cd levels in maternal blood (Liu et al. 2015; Yu et al. 2021). It is also evident that supplementation with calcium and/or zinc, and/or iron can reduce Pb exposure of pregnant and lactating women, fetuses, and children (Ettinger et al. 2007, 2009; Hernandez-Avila et al. 2003; Kordas et al. 2007).

**Fig. 3:**
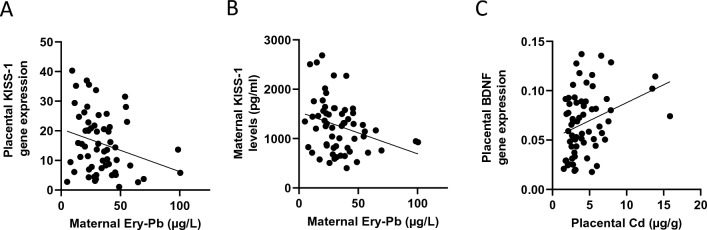
Correlations of maternal Ery-Pb and placental Cd levels and placental gene expression. Scatterplots of **A** maternal serum Ery-Pb and placental KISS-1 gene expression (*N* = 60, *r*_S_ =  − 0.274), **B** maternal serum Ery-Pb and maternal serum KISS-1 levels (*N* = 60, *r*_S_ =  − 0.322), and **C** placental Cd and placental BDNF gene expression levels (*N* = 65, *r*_S_ = 0.328). Gene expression normalized to UBC (Ubiquitin C). *Ery-Pb* erythrocyte lead (Pb) levels

We found that maternal Ery-Pb levels were inversely correlated with maternal serum protein KISS-1 concentrations and with placental gene KISS-1 expression (*P* < 0.05, Fig. 3A, B). In CATREG analysis, only maternal Ery-Pb remained a significant factor of maternal KISS-1 protein levels (Table 3). Placental Cd content correlated positively with placental BDNF gene expression (*P* < 0.05, Fig. 3C). However, in the CATREG analysis with placental BDNF gene expression levels as the dependent variable, placental Cd was no longer a predictive factor (*P* > 0.05, data not shown).

**Table 3:** Factors associated with maternal serum KISS-1 (CATREG analysis)

Factors	*β* ± SE^a^	*P*	Partial *r*	Importance
			[*R*^2^]	Coefficient (rank)
Maternal Ery-Pb (µg/l)	− 0.371 ± 0.103	< 0.001	− 0.371	1.0 (1)
[Crude model, 3 factors, *N* = 38]^b^		< 0.001	[0.333]	
[Final model, 1 factor, *N* = 60]		< 0.001	[0.138]	

^a^Standardized slope ± standard error

^b^The crude model included three variables associated with maternal serum levels in bivariate analysis, i.e., iron supplementation during pregnancy (coded 1 = no supplementation, 2 = multivitamin/multielement supplementation, 3 = intentional iron supplementation); estimated iron intake (mg/day); maternal Ery-Pb levels (µg/l)

Maternal iron status was characterized by various markers, including hemoglobin, serum ferritin, and transferrin saturation (Tf, Table 1). Pregnancy iron deficiency anemia (IDA) in the third trimester is diagnosed by Hb < 11 g/dl and serum ferritin < 30 μg/l (Achebe and Gafter-Gvili 2017; Api et al. 2015; World Health Organization 2008). Maternal iron therapy had no a significant impact on the occurrence of IDA, but improved maternal iron status, as evidenced in the group of pregnant women with higher levels of serum iron, serum ferritin, serum transferrin, and transferrin saturation levels (*P* < 0.05, data not shown). It was also observed that low maternal iron status was associated with low BDNF levels. In regression analysis, maternal iron therapy and Tf saturation were significant modulators of maternal serum BDNF protein levels (Table 4). The models for pro-BDNF and mature BDNF provided the same results (so only the results for pro-BDNF are shown). In addition, we also found that platelet levels were associated with serum BDNF levels (Table 4), indicating the central role of thrombocytes in the regulation of serum BDNF. BDNF release from platelets has been described, but the mechanisms underlying this process have not yet been elucidated (Farmer et al. 2021; Fujimura et al. 2002; Le Blanc et al. 2020), encountering difficulties in interpreting platelet counts in related diseases.

**Table 4:** Factors associated with maternal serum pro-BDNF (CATREG analysis)

Factors	*β* ± SE^a^	*P*	Partial *r*	Importance
			[*R*^2^]	Coefficients (rank)
Maternal iron therapy	0.403 ± 0.145	0.008	0.442	0.423 (1)
Maternal thrombocytes (G/l)	0.414 ± 0.115	< 0.001	0.455	0.334 (1)
Maternal Tf saturation (%)	0.291 ± 0.128	0.027	0.336	0.244 (3)
[Crude model, 7 factors, *N* = 57]^b^			< 0.001	[0.397]
[Final model, 3 factors, *N* = 57]			< 0.001	[0.373]

^a^Standardized slope ± standard error

^b^The crude model included seven factors associated with maternal serum levels in bivariate tests, i.e., parity; years of smoking; iron supplementation during pregnancy (coded 1 = no supplementation, 2 = multivitamin/multielement supplementation, 3 = intentional iron supplementation); maternal mean corpuscular hemoglobin concentration (MCHC, g/dl); maternal thrombocytes (G/l), maternal leukocytes (G/l); maternal transferrin (Tf) saturation (%)

### In Vitro Confirmation of Epidemiological Findings

#### BDNF and KISS-1 Levels of Placental Cells Exposed to Pb and Cd

In order to confirm the findings described above, a series of in vitro experiments were conducted. BeWo cells, a widely used hTC line, were selected and treated with Pb and Cd, respectively, to determine whether metals influence BDNF and KISS-1 expression and secretion in placental cells. Metallothionein 2A (MT2A) is known to be upregulated under conditions of cellular stress exerted by exposure to toxic heavy metals like Cd. We, therefore, also analyzed MT2A gene expression in this cell line.

**Fig. 4:**
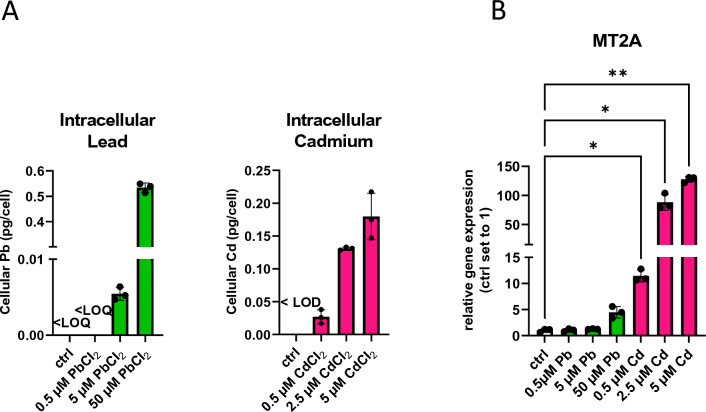
Cellular concentrations of Pb (green) and Cd (pink) of BeWo cells. **A** Intracellular levels of Pb and Cd in BeWo cells exposed for 72 h. **B** mRNA levels of MT2A in cells exposed to Cd and Pb for 72 h. Bar graphs represent mean ± SD from three independent experiments made in technical triplicates. One-Way ANOVA with Welch’s Correction; *P < 0.05, **P < 0.01, ***P < 0.001

Pb and Cd accumulated in BeWo cells (Fig. 4). However, Cd accumulated far more efficiently (34 times more) than Pb, as 5 µM PbCl_2_ results in a mean cellular Pb concentration of 0.005 pg/cell, while equimolar Cd treatment led to a concentration of 0.17 pg/cell (Fig. 4A). Importantly, MT2A was only mildly upregulated by the highest Pb treatment, while it was strongly induced from the lowest Cd dose onwards (Fig. 4B). In addition, Pb treatment reduced cell number and viability, which was accompanied by increased apoptosis and cytotoxicity in a dose-dependent manner (Supplementary Fig. 4). Cd treatment of BeWo cells had no such toxic effect, likely due to the stronger power of Cd in upregulating MT2A, which has been previously described in placental cells (Widhalm et al. 2020). It is furthermore of interest that both Pb and Cd increased total, oxidized, and reduced glutathione (GSH), resulting overall in a decreased GSH/GSSG ratio, an ultimate indicator of cellular oxidative stress (Supplementary Fig. 5).

**Fig. 5:**
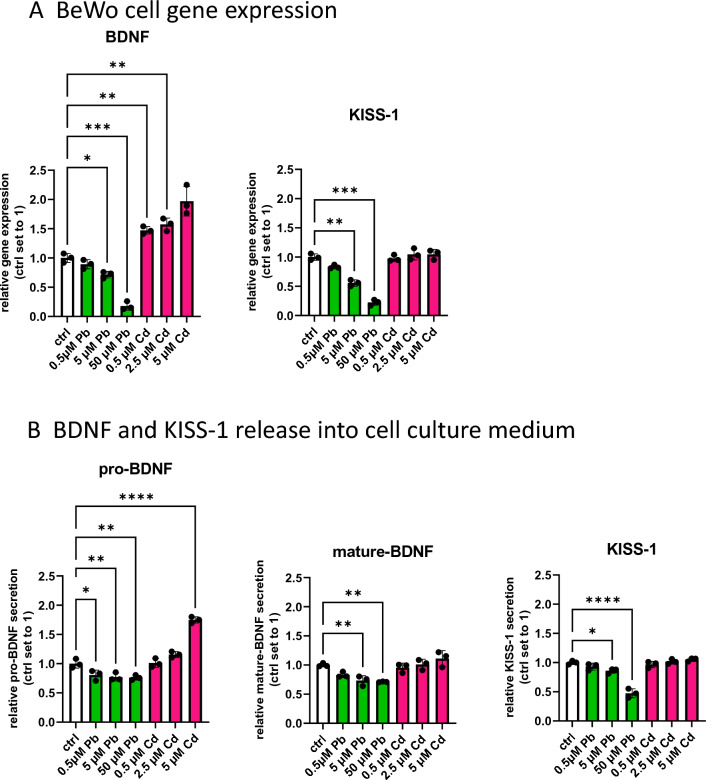
BeWo cell gene expression and release of BDNF and KISS-1 upon Pb or Cd treatment. **A** mRNA levels of BDNF and KISS-1 in BeWo cells treated with indicated Pb and Cd concentrations for 72 h. **B** Secreted pro-BDNF, mature BDNF, and KISS-1 in cell culture medium of BeWo cells treated for 72 h with Pb or Cd. Bar graphs represent mean ± SD from three independent experiments made in technical triplicates; One-Way ANOVA with Welch’s Correction; *P < 0.05, **P < 0.01, ***P < 0.001

Pb-exposed BeWo cells showed decreased BDNF and KISS-1 expression (Fig. 5A), consistent to some extent for KISS-1 with the finding that maternal Ery-Pb remained the only factor associated with maternal KISS-1 levels (Table 3). Moreover, Pb-treated BeWo cells showed reduced release of pro and mature BDNF as well as of KISS-1 (Fig. 5B). To our knowledge, a relationship between Pb and KISS-1 has not yet been described. Pb, however, is well known to interfere with BDNF signaling by inhibition of N-methyl-d-aspartate (NMDA) receptor and, therefore, has been suggested as effect biomarker for Pb-induced neurodevelopmental toxicity (reviewed by Gundacker et al. 2021a, b). Although the molecular function of BDNF in the human placenta is not fully understood, BDNF is expressed in this tissue and seems to be important for placental development (Mayeur et al. 2010). Our in vitro data on placental cells confirmed to some extent that Pb has the potential to alter BDNF expression and release in the human placenta. The underlying mechanisms need to be elucidated in future studies.

Cd exposure during prenatal and early-child phase is associated with impaired neurodevelopment and deteriorations in motoric and cognitive function in children (Chatterjee and Kortenkamp 2022; Ciesielski et al. 2012; Wang et al. 2016a). Its neurotoxic effects include generation of oxidative stress by ROS formation and impairing antioxidant defense mechanisms, induction of cell death, inflammatory responses, disrupting the release of neurotransmitters (e.g., glutamate), reducing the expression of proneuronal genes, and epigenetic alterations (Gonçalves et al. 2021; Wang and Du 2013). Among the proneuronal genes that are affected by Cd, it is also BDNF. Cd levels of mothers were negatively associated with BDNF serum levels, and developmental quotients in their offspring (Wang et al. 2016b). Furthermore, adolescent males with higher Cd body burdens had increased CpG methylation at blood BDNF DNA and reduced BDNF serum levels (Rodriguez-Carrillo et al. 2022b).

In good agreement with the finding that placental Cd was associated with placental BDNF expression in our group of neonates (Fig. 3C), Cd-treated BeWo cells showed increased BDNF expression (Fig. 5A). Further discrimination between the different BDNF isoforms by ELISA revealed that the metal specifically upregulated pro-BDNF levels (Fig. 5B). It is conceivable that Cd in treated cells displaces zinc (Zn) from proteins and enzymes and thereby increases the labile Zn pool (Day et al. 1984; Urani et al. 2015). Zn in turn is known to induce BDNF mRNA expression (Hwang et al. 2011). As matrix metalloproteinase (MMP)2 and MMP9, which are involved in the formation of mature BDNF from pro-BDNF, are inhibited by Cd (Lacorte et al. 2015), this could explain, why mature BDNF levels were not upregulated by Cd exposure. Further studies should determine whether the increase in pro-BDNF, known for its pro-apoptotic features, adversely affects placental cells. This highlights that, although the placenta efficiently retains Cd, thereby protecting the fetus from direct exposure, the metal can accumulate in the placenta, potentially inhibit proper placental function, thereby having secondary adverse outcomes for fetal (neuro)development (Kippler et al. 2010; Liu et al. 2022; Vilahur et al. 2015).

#### BDNF and KISS-1 Levels of Placental Cells Under Iron Deficiency and Iron Overload Conditions

Since we observed that iron supplementation during pregnancy is an important factor associated with maternal serum pro-BDNF, mature BDNF, and to some degree also KISS-1 concentrations, which correlate well with placental BDNF and KISS-1 levels, we aimed to experimentally confirm the influence of iron status on BDNF and KISS-1 release from trophoblast cells.

**Fig. 6:**
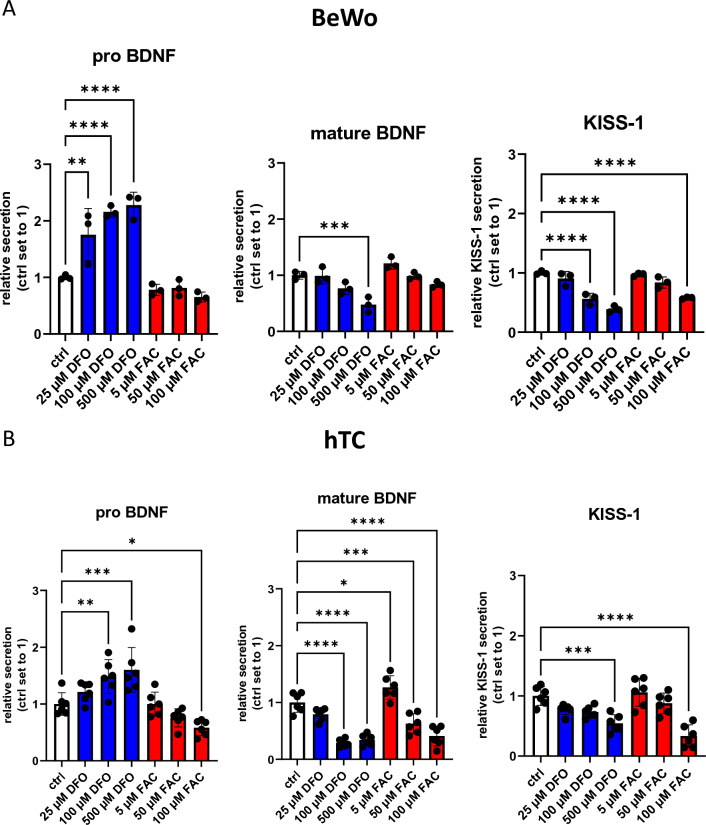
Release of BDNF and KISS-1 from BeWo cells and hTCs under iron deficiency (blue) and iron overload (red) conditions. Secretion of pro-BDNF, mature BDNF, and KISS-1 after treatment of BeWo (**A**) and hTCs (**B**) with DFO (iron deficiency condition) and FAC (iron overload condition). Data from three (BeWo) or six independent experiments (hTCs isolated from six individual placentas) are shown. One-Way ANOVA with Welch´s Correction; *P < 0.05, **P < 0.01, ***P < 0.001

In a first step, BeWo cells were treated with DFO (iron deficiency condition) or FAC (iron overload condition) in order to determine the release of pro-BDNF, mature BDNF, and KISS-1 into medium, analyzed by ELISA. After DFO treatment, the secretion of pro-BDNF into cell culture medium increased in a dose-dependent manner, while mature BDNF secretion decreased (Fig. 6A). Furthermore, a significant decrease of KISS-1 secretion in the medium was observed upon treatment with 500 µM of DFO (Fig. 6A). In contrast, FAC treatment led to a dose dependent, but non-significant reduction of pro-BDNF, mature BDNF, and KISS-1. These findings were confirmed in hTCs that responded in a well-comparable manner as BeWo cells (Fig. 6B). Importantly, choriocarcinoma-derived BeWo cells seemed to cope better with iron overload conditions, which could stem from the higher iron demands of cancer cells (Manz et al. 2016).

Studies in rodents have shown that iron deficiency leads to downregulation of BDNF expression in the hippocampus without compensatory upregulation of its specific receptor (tyrosine receptor kinase B) (Tran et al. 2008), furthermore that iron deficiency can cause developmental deficits by reducing the expression and function of IGF-I/II and BDNF in specific areas of the brain (Estrada et al. 2014). Such findings are in accordance with our in vitro experiments, where iron deficiency led to a decreased release of mature BDNF from BeWo cells. Notwithstanding, we have no explanation yet for the higher pro-BDNF and the lowered KISS-1 release from BeWo cells and what this would mean for proper placental function.

#### Strengths and Limitations of the Study

The present pilot study included 65 mother–newborn pairs. The small sample size implies the need for validation of our findings in larger follow-up studies to test the predictive potential of BDNF and KISS on reproductive and neurodevelopmental health. Limited financial resources prevented to also measure other chemicals known to interfere with BDNF levels such as BPA (Mustieles et al. 2022), phthalates (Ponsonby et al. 2016), arsenic (Pandey et al. 2017), or some pesticides (Rodriguez-Carrillo et al. 2022a) among others. Future studies could also investigate the gene variants of proteins involved in oxidative stress defense and iron metabolism [e.g., MT2A, hemochromatosis (HFE), transferrin] that may modulate the relationships observed here. We have confirmed central findings of the epidemiological study in in vitro experiments using, among others, human primary placental cells. The hybrid multidisciplinary approach, combining an epidemiological study with in vitro experiments, i.e., direct experimental testing of associations observed in human studies, is a clear strength of this study. Other strengths include the inclusion of susceptibility biomarkers (iron status parameters) and the measurement of both pro- and mature BDNF isoforms.

## Conclusions

Our study demonstrates how exceptionally well maternal BDNF and KISS-1 levels are correlated with both placental gene expression and cord serum protein levels. Therefore, the maternal levels have a great potential to become validated markers of placental health and fetal (neuro)development. Interpretation of our results on KISS-1, including the in vitro results, is difficult because there are virtually no comparative data. However, our data indicate that KISS-1 is not only important at early stages of pregnancy, such as for implantation. KISS-1 levels could be regulated by maternal iron levels and/or prenatal Pb exposure, which should be the subject of further studies.

Overall, the observed associations need to be confirmed in a larger sample and validated in terms of placental, reproductive, and neurodevelopmental function. The relationships between placental BDNF levels, maternal iron status, and prenatal Pb exposure, and how their interplay affects brain development, remain to be explored.

## Supplementary Information

Below is the link to the electronic supplementary material.

Supplementary file1 (DOCX 324 kb)

## Data Availability

The data supporting the results of this study are available upon request from the corresponding author (Claudia Gundacker).
