# Supplementary material for: BDNF and KISS-1 Levels in Maternal Serum, Umbilical Cord, and Placenta: The Potential Role of Maternal Levels as Effect Biomarker
Source: Expo Health. 2023 May 29:1–17. Online ahead of print. doi: 10.1007/s12403-023-00565-w (PMC10225291; doi:10.1007/s12403-023-00565-w)
Supplement: Supplementary file 1 — Supplementary file1 (DOCX 324 kb) [file 12403_2023_565_MOESM1_ESM.docx]

**Supplementary Information**

**BDNF and KISS-1 levels in maternal serum, umbilical cord and placenta: The potential role of maternal levels as effect biomarker**

Sebastian Granitzer^1,6 #^, Raimund Widhalm^1,6 #^, Simon Atteneder^1^, Mariana F. Fernandez^2,3,4^, Vicente Mustieles^2,3,4^, Harald Zeisler^5^, Markus Hengstschläger ^1^, Claudia Gundacker^1,6^

^1^Medical University of Vienna, Institute of Medical Genetics, Vienna, Austria

^2^University of Granada, Center for Biomedical Research (CIBM), Granada, Spain

^3^Instituto de Investigación Biosanitaria (ibs.GRANADA), Granada, Spain

^4^Consortium for Research and Public Health (CIBERESP), Instituto de Salud Carlos III, Madrid, Spain

^5^ Medical University Vienna, Department of Obstetrics and Gynecology, Vienna, Austria

^6^Exposome Austria, Research Infrastructure and National EIRENE Hub, Austria

^#^ These authors contributed equally to this work

**Corresponding Author**

Claudia Gundacker

Institute of Medical Genetics, Medical University of Vienna

Waehringer Strasse 10, A-1090 Vienna, Austria

Tel: +43 1 40160 56503

E-mail: [claudia.gundacker@meduniwien.ac.at](mailto:claudia.gundacker@meduniwien.ac.at)


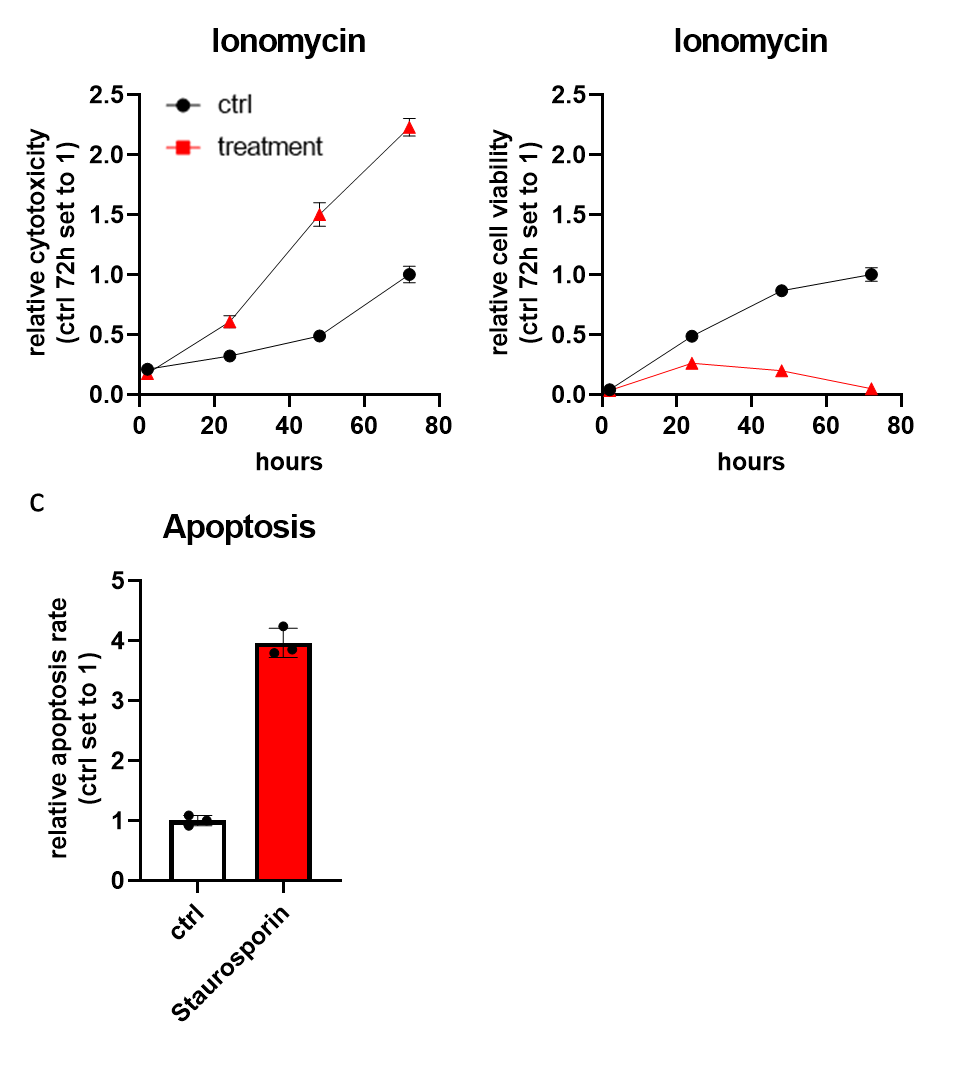


**Suppl. Figure 1. Positive controls** of cytotoxicity (a), viability (b) and apoptosis (c) assays according to Suppl. Figure 4. (N=3)


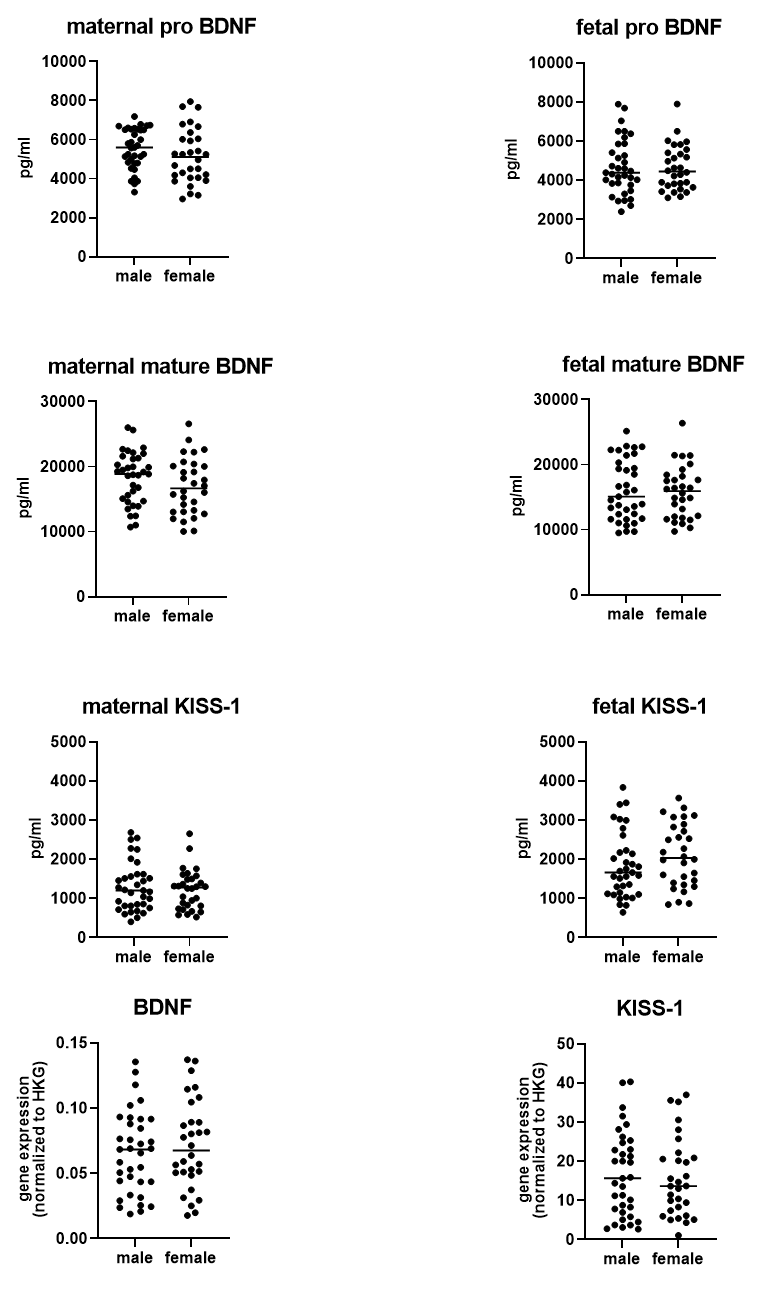


**Suppl. Figure 2.** No sex specific differences in serum levels and gene expression of BDNF and KISS-1 (35 male and 30 female newborns).


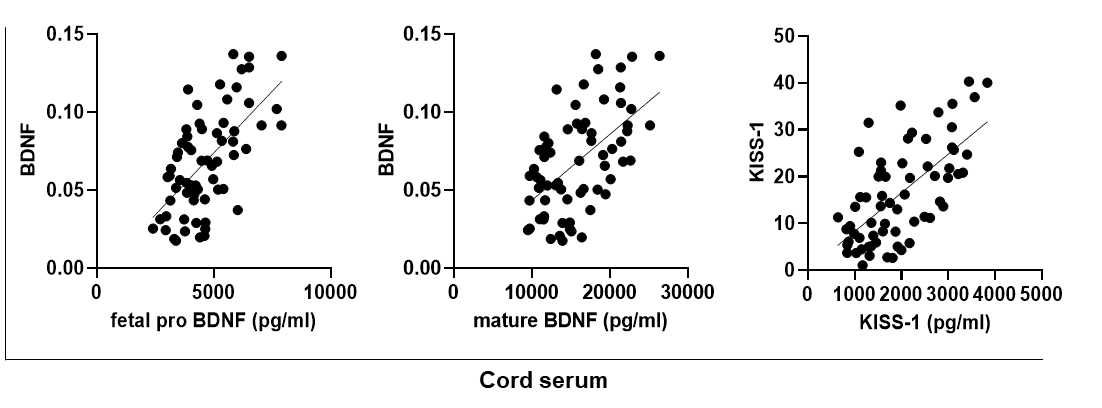


**Suppl. Figure 3**. Cord serum levels of pro BDNF, mature BDNF, and KISS-1 correlate with placental gene expression levels (N=65).


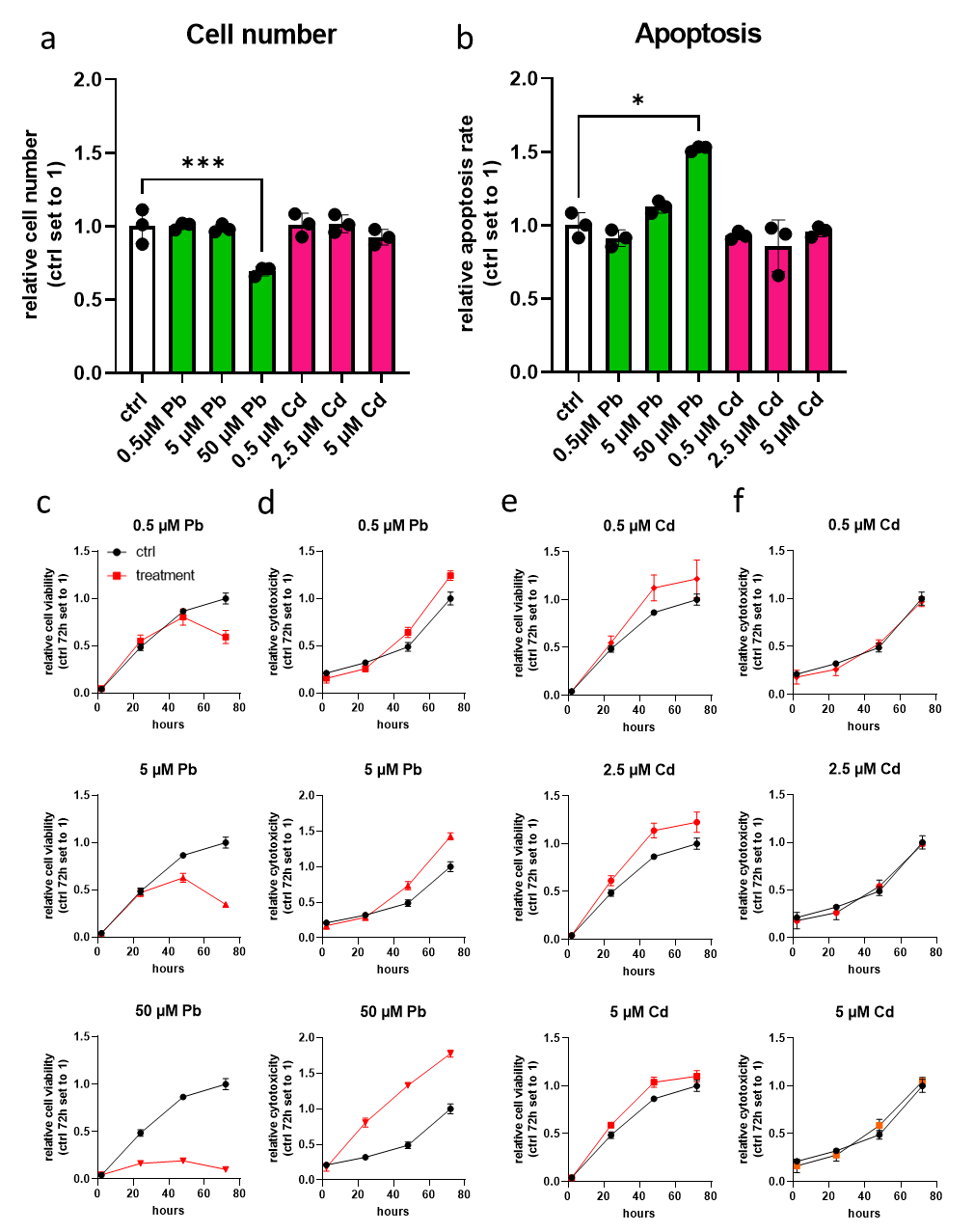


**Suppl. Figure 4. Cell number (a), apoptosis (b), cell viability (c, e), and cytotoxicity (d, f) in Pb and Cd treated BeWo cells.** Bar graphs represent mean ± SD from three independent experiments made in technical triplicates; One-Way ANOVA with Welch´s Correction; *P<0.05,***P<0.001


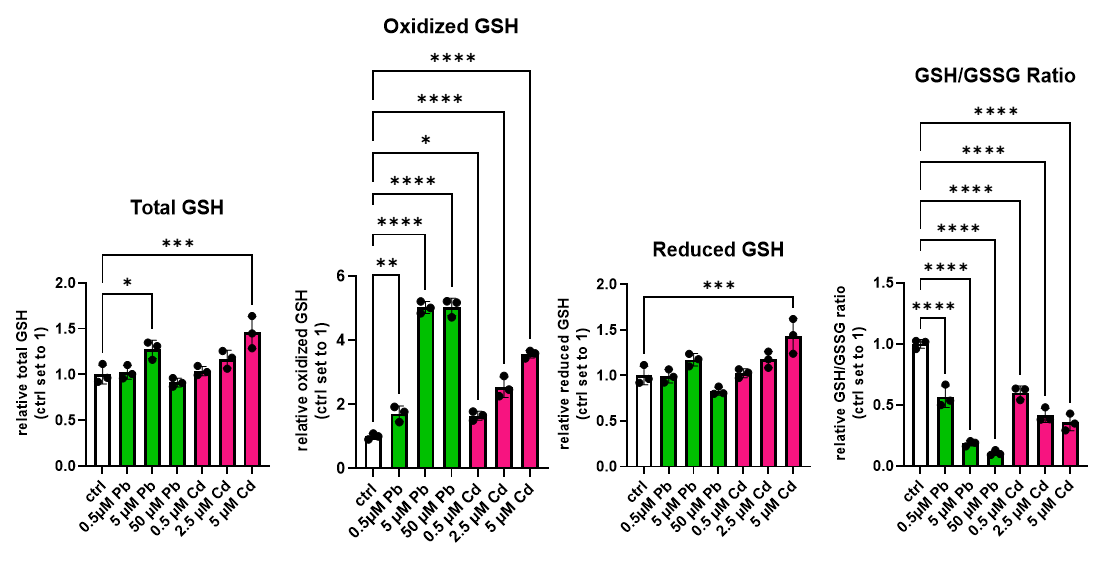
**Suppl. Figure 5. Glutathione status of Pb and Cd treated BeWo cells**. Bar graphs represent mean ± SD from three independent experiments made in technical triplicates; One-Way ANOVA with Welch´s Correction; *P<0.05, **P<0.01, ***P<0.001, ****P<0.0001
